# Dual diagnosis of trisomy 21 and lethal perinatal Gaucher disease as a cause of non‐immune hydrops fetalis in a twin pregnancy for a consanguineous couple

**DOI:** 10.1002/ccr3.7827

**Published:** 2023-08-23

**Authors:** Talib Al Harthy, Samantha Colaiacovo, Robert J. Gratton, Kevin Coughlin, Victoria Mok Siu, Chitra Prasad, Charles Rupar, Maha Saleh

**Affiliations:** ^1^ Schulich School of Medicine and Dentistry Western University London Ontario Canada; ^2^ Division of Pediatric Neonatology, Department of Pediatrics London Health Sciences Center London Ontario Canada; ^3^ Division of Clinical Genetics, Department of Pediatrics London Health Sciences Center London Ontario Canada; ^4^ Department of Obstetrics & Gynecology London Health Sciences Center London Ontario Canada; ^5^ Department of Pathology and Laboratory Medicine London Health Sciences Center London Ontario Canada

**Keywords:** down syndrome, dual diagnosis, gaucher, hydrops, NIPT

## Abstract

Non‐immune hydrops is a prenatal finding which can occur due to an underlying genetic diagnosis such as common chromosomal aneuploidy (Trisomy 21, Turner syndrome etc.). It is extremely rare to have more than one genetic cause of hydrops fetalis in a single pregnancy. This report describes a dichorionic diamniotic pregnancy for a consanguineous couple where noninvasive prenatal testing was “high risk” for Trisomy 21. Family declined amniocentesis and opted for postnatal genetic testing. The pregnancy was later complicated with severe hydrops fetalis leading to demise for one of the twins, and a premature delivery of the other twin who had remarkable collodion not in keeping with Trisomy 21. Postnatal genetic investigations confirmed both Trisomy 21 and prenatal lethal Gaucher disease in the survivor twin. This case report highlights some of the prenatal diagnostic challenges for a consanguineous couple where a rare cause of fetal hydrops was concealed in a setting of a common chromosomal aneuploidy. The prompt postnatal diagnosis of perinatal lethal Gaucher disease, confirmed with undetectable glucocerebrosidase enzyme activity, assisted the family in the decision of providing palliative care for their infant who was quickly deteriorating. The importance of postnatal genetic evaluation and its impact on immediate patient management in an NICU setting is emphasized. This dual diagnosis was significant for the couple as it explained pervious pregnancy losses and has important future recurrence risk implications.

## Graphical abstract

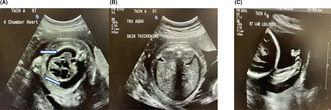

## 1 BACKGROUND

Hydrops Fetalis (HF) is one of the complex problems encountered by Maternal Fetal Medicine (MFM) and Perinatal Medicine with an incidence range from 1/1700 to 1/3000^1^, ^2^ HF typically results from pathological accumulation of fluid in various fetal tissue spaces including the skin and at least one of the three body cavities: pleural, pericardial, and peritoneal.^3^ Based on etiology, HF is classified into two types: Immune Hydrops Fetalis (IHF) and NonImmune Hydrops Fetalis (NIHF).

IHF is less common and accounts for about 10% of HFs. It results from alloimmune processes which lead to severe fetal anemia, ultimately causing fetal hydrops.

In contrast to IHF, NIHF accounts for 90% of all HFs.^4^ NIHF includes a wide range of different diagnoses such as congenital infection, nonimmune fetal anemia, congenital malignancy, isolated congenital anomalies (e.g., heart defect, renal anomalies) and other genetic and metabolic causes.^5^

Among the genetic causes, chromosomal abnormalities such as Trisomy 21,13, and18, and Monosomy X (Turner Syndrome) are the most common genetic causes of NIHF. Collectively, those chromosomal abnormalities account for 7%–16% of NIHFs.^4^, ^5^ Trisomy 21 is the second most common aneuploidy leading to NIHF after Turner syndrome and accounts for 23%–30% of the chromosomal cases. Other genetic causes include single gene disorders associated with inborn errors of metabolism and other rare autosomal recessive Mendelian disorders which together account for 1%–2% of NIHF.^6^

Prenatally, once HF is identified, close follow up with MFM is recommended to determine the cause of HF and direct appropriate interventions. Once IHF is ruled out, a stepwise approach is followed to explore the cause of NIHF. The spectrum ranges from lethal to nonlethal or treatable causes. Identifying a cause can help pregnant women make informed decisions with regards to options such as pregnancy termination or interventions to improve fetal survival such as in utero fetal blood transfusions, selected mode or place for delivery.^6^, ^7^

This stepwise approach to explore the cause of NIHF entails excluding common TORCH infections, fetal anemia, fetal cardiovascular concerns, as well as common chromosomal aneuploidies.^8^ If initial workup fails to identify a common cause, further genetic testing may be required. Currently, NIHF gene panels are available. These next generation sequencing panels include several genes linked to inborn errors of metabolism and other rare autosomal recessive Mendelian disorders that have been reported in association with NIHF.^9^

In this paper we share a case where two genetic etiologies of NIHF coexisted in the same patient. This case report highlights some of the prenatal diagnostic challenges for a consanguineous couple where a rare cause of fetal hydrops was concealed in a setting of a common chromosomal aneuploidy.

## 2 CASE REPORT

**FIGURE 1:**
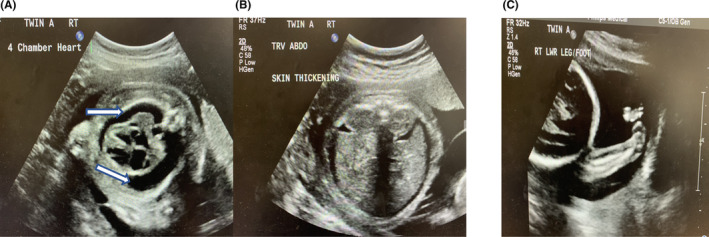
Twin A: (A) Bilateral pleural effusion, (B) moderate ascites and skin thickening indicating hydrops fetalis. Amniotic fluid is normal. (C) Right club foot.

Our patient is a 38‐ year‐ old G7, T3, A3 healthy woman of Middle Eastern heritage with a history of recurrent miscarriages and unexplained pregnancy losses. Her family history was remarkable for consanguinity and no other known genetic diagnoses. She presented to a regional multidisciplinary prenatal clinic with abnormal anatomy ultrasound findings for an unplanned diamniotic dichorionic twin pregnancy. A 22‐week anatomy scan showed bilateral clubfeet, choroid plexus cyst, and a cardiac left ventricular echogenic focus in Twin B. Right sided clubfoot was identified in twin A (Figure 1). Given those findings, suspicion for a chromosomal diagnosis was high. Options for investigating this further were discussed with the couple including noninvasive prenatal testing (NIPT) for common aneuploidy as well as invasive testing via amniocentesis. They opted for NIPT which returned as high risk for Trisomy 21 for the twin pregnancy. The couple was also offered G‐banded chromosome karyotype for themselves given the history of recurrent miscarriages, with results showing no evidence of a balanced structural rearrangement of chromosomes.

The couple were counseled for the high likelihood of Trisomy 21. However, they deferred confirmatory amniocentesis and opted for postnatal testing. Fetal echo and a follow‐up ultrasound were coordinated.

**FIGURE 2:**
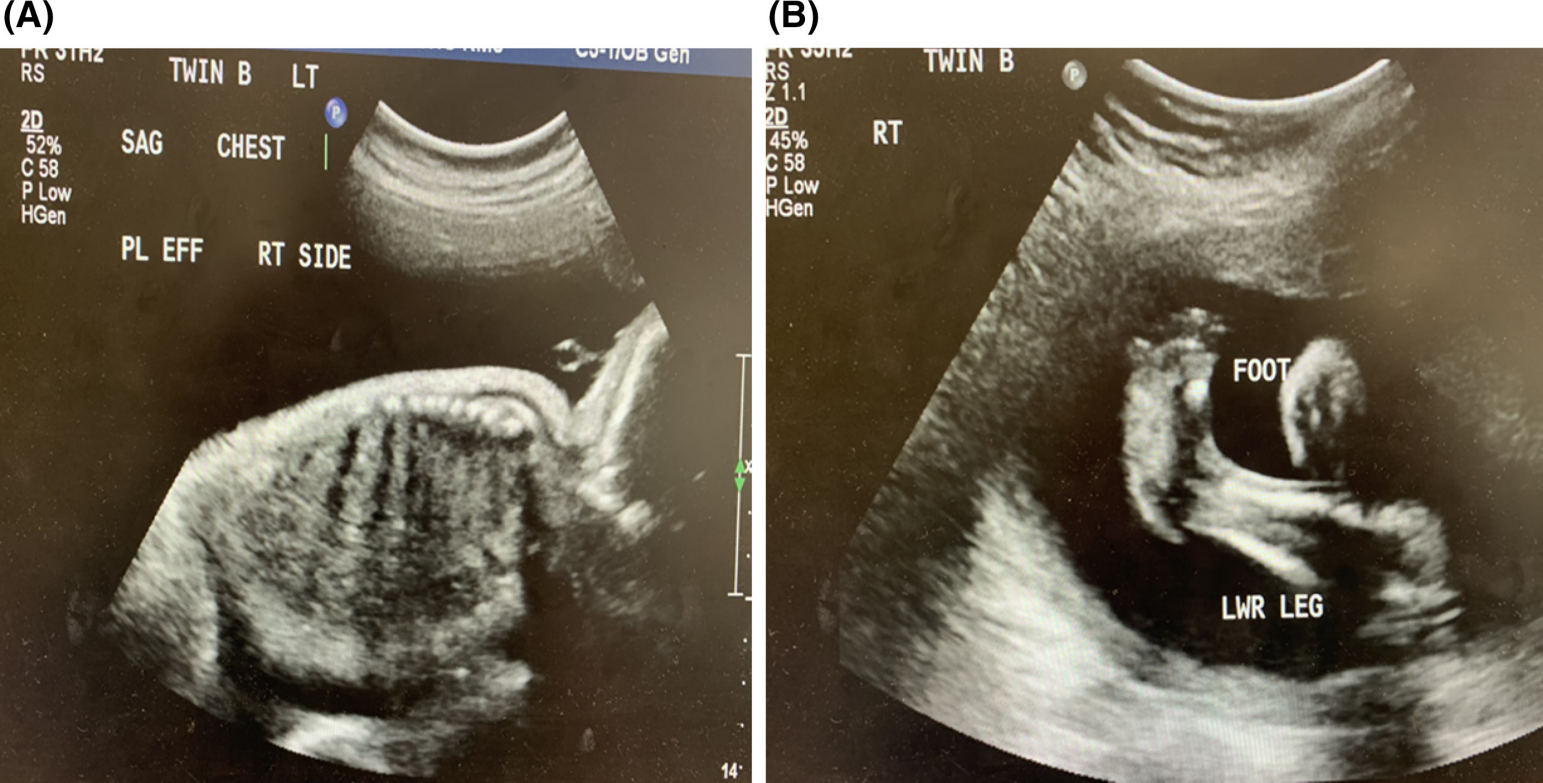
(A) Twin B Right sided pleural effusion. (B) Clubbed foot, rocker bottom appearance.

Subsequent follow up ultrasounds at 28 weeks showed bilateral pleural effusions in twin A and right sided pleural effusion in twin B. (Figure 2) Later ultrasounds confirmed hydrops fetalis for both twins. There was no sonological evidence of fetal anemia and TORCH screen was negative. At this point the hydrops fetalis was attributed to Trisomy 21.

At 31 weeks of gestation, emergency cesarean section was indicated due to abnormal umbilical doppler in twin A. Magnesium sulphate was given for neuroprotection, yet there was no time to administer prenatal steroids.

Twin A was born with no respiratory effort, cyanosis and a heart rate less than 60 beats per minute. Due to laryngeal edema, multiple attempts of intubation failed. A laryngeal mask was inserted. Heart rate remained less than 60 beats per minute despite adequate ventilation, therefore, chest compressions were initiated, followed by intravenous epinephrine. Twin A was declared dead after 48 min of unsuccessful resuscitation. Physical examination showed weight of 1660 grams (47% percentile), male genitalia, and massive edema. The sample for DNA banking was not collected and parents declined fetal autopsy.

Twin B was also born with poor respiratory effort and poor tone, and had Apgar Scores of 1,3,4 at 1, 2 and 10 min of life. The baby required positive pressure ventilation and was eventually intubated. Arterial cord gas was pH of 7.27 with a base deficit of 5.2; Venous cord gas pH of 7.23 and base deficit of 4.5.

On physical examination his weight was 1340 g (24th percentile); head circumference was 28 cm (38th percentile; and length was 37 cm (8th percentile). The baby looked edematous with shiny, scaly and tight overall skin. A collodion membrane was noted around the fingers. A third fontanelle was palpable. His facial features were not typical for Trisomy 21 but he did have low set ears and upslanting palpebral fissures. The eyes were constantly open (ectropion). He had a depressed nasal root with normal appearance of the lips without eclabium. His neck was short and broad. There was full range of movement at the elbows. He had bilateral camptodactyly with absent distal interphalangeal creases in all fingers, and visible bilateral single transverse palmar creases. His hips were constantly extended. There were mild flexion contractures at the knees bilaterally, a fixed left talipes equinovarus, and a malleable right talipes equinovarus. He had male external genitalia with bilateral cryptorchidism.

The postnatal clinical course of twin B was complicated by respiratory failure requiring high frequency ventilation, hypotension, and subsequent acute kidney injury. He developed anemia, thrombocytopenia, coagulopathy, and hypoalbuminemia for which he required several transfusions including packed red cell, platelets, fibrinogen, and albumin.

Postnatal sonological assessment showed bilateral grade 1 intraventricular hemorrhage. Normal abdominal and renal ultrasounds. His echocardiogram showed large patent ductus arteriosus (PDA), a small secundum atrial septal defect (ASD) or patent foramen ovale (PFO) and some findings suggestive of persistent pulmonary hypertension (PPHN).

The rapid aneuploidy test via quantitative fluorescence polymerase chain reaction (QF‐PCR) confirmed Trisomy 21 in a male baby. G‐band analysis confirmed a nondisjunction. Given the collodion membrane, ectropion (which is atypical of Trisomy 21) as well as the parental consanguinity, a dual diagnosis was proposed. Chitotriosidase levels were assayed as a screen for lysosomal storage disorders, but results were 0 nmol/hr/ml, thus this could not be used as a screen. Perinatal lethal Gaucher disease was suspected which was confirmed through glucocerebrosidase enzyme activity of 0.3 nmoL/hr/mg protein (reference level 5.0 to 11.3).

Based on this enzyme activity, parents were counseled about Gaucher disease‐ perinatal lethal type. The couple chose to withdraw care and apply comfort measures. The baby was extubated on day 10 of life and died peacefully in his family's presence.

Postmortem pathology was offered but declined by the family. The molecular confirmation was later provided through lysosomal storage disorders next generation sequencing panel which identified a homozygous pathogenic variant in the glucocerebrosidase (GBA) gene: GBA:c.1448 T > C, p.(Leu483Pro). This variant is associated with an autosomal recessive form of Gaucher disease (OMIM # 608031, 230,800, 230,900, 231,000, 231,005). The parents were confirmed to be carriers for this variant.

## 3 DISCUSSION

NIHF is one of the complex problems encountered prenatally. It is impossible to pinpoint an etiology based on ultrasound findings alone. It is therefore important to follow a stepwise approach to explore the cause of NIHF.

In our case the high‐risk result for Trisomy 21 on NIPT was in keeping with the early anatomy finding of clubfeet, echogenic cardiac foci and choroid plexus cyst that have been reported with that common trisomy.^10^, ^11^, ^12^ This diagnosis was also felt to explain the NIHF. Had the couple opted for an amniocentesis, a confirmation of Trisomy 21 on a prenatal QF‐PCR might have precluded additional testing through next generation sequencing panels that cover genes linked to NIHF or inborn errors of metabolism including storage disorders such as Gaucher Disease.

Gaucher Disease—perinatal lethal type was suspected after birth due to additional clinical exam finding of skin abnormalities which was not explained by the Trisomy 21. This diagnosis of Gaucher Disease was important for this couple who had already lost one twin and had a critically ill baby in the NICU setting. With the finding of a lethal genetic disorder, they could now understand the reason for their previously unexplained pregnancy losses, one of which also had HF. The suspicion of a specific diagnosis enabled rapid confirmation with enzyme analysis rather than waiting for next generation sequencing panel or whole exome sequencing which would have delayed a definitive diagnosis by weeks. Based on the results, definitive management decisions could be made. As well, this information was important given a 25% recurrence risk for Gaucher disease. Early prenatal diagnosis in subsequent pregnancies and carrier testing for first degree relatives of child bearing age was now also possible.

Based on literature reviews, there is only one reported prenatal case with a dual diagnosis of Gaucher disease and Down Syndrome. This fetus was diagnosed prenatally as chorionic villus sampling was done based on a known family history of Gaucher disease and a known familial variant. The Trisomy 21 in that case was confirmed on a QF‐PCR as part of a first‐tier prenatal sample test analysis. That pregnancy was accordingly terminated.^13^ In our case the couple opted to continue with the pregnancy which gave us the opportunity to examine the babies and document those physical findings. Thus, we believe that our case is the first case to report the postnatal examination findings of a live birth with dual genetic and metabolic diagnosis.

## 4 CONCLUSION

In this case, we report a dual genetic diagnosis of Trisomy 21 and prenatal lethal Gaucher disease as a cause of NIHF in a twin pregnancy. This case highlights the importance of extensive genetic evaluation with high index of suspicion of more than one genetic etiology for NIHF, especially in cases of consanguinity and recurrent unexplained miscarriages.

## AUTHOR CONTRIBUTIONS

**Talib Al Harthy:** Data curation; formal analysis; writing – original draft. **Samantha Colaiacovo:** Writing – review and editing. **Robert J. Gratton:** Writing – review and editing. **Kevin Coughlin:** Writing – review and editing. **Victoria Mok Siu:** Writing – review and editing. **Chitra Prasad:** Writing – review and editing. **Charles Rupar:** Methodology; writing – review and editing. **Maha Saleh:** Data curation; writing – original draft; writing – review and editing.

## CONFLICT OF INTEREST STATEMENT

All authors declare no conflict of interest.

## ETHICS STATEMENT

The local Institutional Review Board deemed the study exempt from review.

## CONSENT

Written informed consent was obtained from the patient to publish this report in accordance with the journal's patient consent policy.

## Data Availability

None
